# Knowledge and Behaviors Toward COVID-19 Among US Residents During the Early Days of the Pandemic: Cross-Sectional Online Questionnaire

**DOI:** 10.2196/19161

**Published:** 2020-05-08

**Authors:** John M Clements

**Affiliations:** 1 Master of Public Health Program Division of Public Health, College of Human Medicine Michigan State University Flint, MI United States

**Keywords:** public health, surveillance, COVID-19, knowledge, behavior, outbreak, infectious disease, health information

## Abstract

**Background:**

The early days of the coronavirus disease (COVID-19) pandemic in the United States brought uncertainty in the knowledge about COVID-19 and what to do about it. It is necessary to understand public knowledge and behaviors if we are to effectively address the pandemic.

**Objective:**

The aim of this study is to test the hypothesis that knowledge about COVID-19 influences participation in different behaviors including self-reports of purchasing more goods than usual, attending large gatherings, and using medical masks.

**Methods:**

This study was funded and approved by the Institutional Review Board on March 17, 2020. The cross-sectional online survey of 1034 US residents aged 18 years or older was conducted on March 17, 2020.

**Results:**

For every point increase in knowledge, the odds of participation in purchasing more goods (odds ratio [OR] 0.88, 95% CI 0.81-0.95), attending large gatherings (OR 0.87, 95% CI 0.81-0.93), and using medical masks (OR 0.56, 95% CI 0.50-0.62) decreased by 12%, 13%, and 44%, respectively. Gen X and millennial participants had 56% and 76% higher odds, respectively, of increased purchasing behavior compared to baby boomers. The results suggest that there is a politicization of response recommendations. Democrats had 30% lower odds of attending large gatherings (OR 0.70, 95% CI 0.50-0.97) and 48% lower odds of using medical masks (OR 0.52, 95% CI 0.34-0.78) compared to Republicans.

**Conclusions:**

This survey is one of the first attempts to study determinants of knowledge and behaviors in response to the COVID-19 pandemic in the United States. A national, coordinated effort toward a pandemic response may ensure better compliance with behavioral recommendations to address this public health emergency.

## Introduction

Some of the most important problems in the world require an understanding and acceptance of science by the general public, including addressing health problems such as the emergence of the novel coronavirus (severe acute respiratory syndrome coronavirus 2 [SARS-Cov-2]) and subsequent disease (coronavirus disease [COVID-19]) transmission. SARS-CoV-2 first emerged in December 2019 in Hubei Province in Wuhan, China [1]. By mid-January 2020, Thailand and Japan were the first countries outside of China to report COVID-19 cases [1]. The Chinese government subsequently quarantined the greater Wuhan area on January 23, 2020, to prevent COVID-19 spread [2].

On January 21, 2020, the first COVID-19 case in the United States was reported in Washington State [3], and it was later reported that public health officials thought the virus was prevalent in the community for at least several weeks [4]. In the United States, the federal government ordered that certain flights from China be halted and passengers from other locations at different ports of arrival would be screened [5]. The Centers for Disease Control and Prevention (CDC) and the National Institutes of Health (NIH) began making recommendations based on the scientific knowledge of the situation to limit social contacts, encourage wise use of medical supplies including masks, and assure the public about the reliability of the food and consumable goods supplies [6]. However, even after these recommendations, there were reports of college students waiting in long lines at bars to celebrate their campuses closing [7], people buying medical-grade masks [8], and people hoarding everything from toilet paper to eggs and milk [9], even as the President sought to reassure the public that the supply of food and goods was secure [10].

Scholarship on the public understanding of science (PUS) aims to explain public understanding of, involvement in, and trust in science. In the face of the current pandemic, this requires the public to understand and trust those who are making recommendations to limit exposure and the spread of the illness. The deficit model of PUS posits that a lack of support for science (and a subsequent rejection of recommendations) is due to a lack of understanding about science, and if scientists can find a way to fill this knowledge deficit, then support for science will increase. A more contemporary view of PUS is that the public’s knowledge is not deficient, but rather there is a deficit in trust of science and in scientific experts specifically. Because of an increasing lack of trust in these institutions, Solomon [11] observed that there is an increased personal rejection of science, which then leads to lower levels of scientific literacy and understanding of science. Low literacy and understanding may influence people to not follow recommendations for addressing science-based problems as is evident with the current pandemic.

Much of the PUS literature examines trends in scientific knowledge (albeit self-reported knowledge for the most part) and attitudes about science. Results are mixed as to whether increased knowledge leads to positive attitudes (variously described as trust, support, confidence, and support for funding) about science. Allum et al [12] observed a small positive correlation between knowledge about science and positive attitudes about science, and Miller [13] reports that there is public support for science even in the face of a scientific literacy rate of 20%. The public’s support for science is necessary when addressing many important social issues, including an immediate need for the public to understand and trust the science about the novel coronavirus pandemic currently plaguing the world. If the public does not trust the underlying science about these issues and does not trust institutions that are tasked with managing this threat, it will be difficult to count on public support for policies to address these issues.

This paper describes a cross-sectional online survey designed to gauge public knowledge and behaviors about COVID-19 in the United States. Zhong et al [14] conducted a similar study in China, approximately 1 week after the Hubei Province was put on lockdown (approximately 8 weeks after the first case emerged), to determine the level of knowledge and public sentiment about the emerging pandemic in China. This study essentially replicates questions about knowledge from that study while asking about more specific behaviors. The sample was drawn from an online work platform (Amazon’s Mechanical Turk) to determine the level of knowledge about COVID-19 and characteristics that influence knowledge and behaviors toward COVID-19. This is among one of the first attempts to investigate determinants of knowledge and behaviors in the public related to COVID-19 in the United States.

The general hypothesis guiding this research is that lower levels of knowledge about the coronavirus pandemic are associated with behaviors that are contrary to current guidelines that suggest against panic buying, large gatherings, and the use of medical masks. Furthermore, there are differences in knowledge and behaviors in different age groups, sex, education level, race, income, and political party identification.

## Methods

### Participants

This cross-sectional study recruited a convenience sample of respondents from Amazon Mechanical Turk (MTurk). MTurk is an online platform for recruiting remote workers to complete small tasks for small amounts of money. Some studies report that MTurk sample demographics are closer to the US general public than typical university samples [15,16] and tend to be more diverse than other internet samples [17]. MTurk provides a quick, inexpensive method to collect data from a wide cross-section of the general public.

The MTurk interface allows requestors (author JC) to advertise human intelligence tasks (ie, the survey in this case) to workers (survey participants). Although the survey was included on a website that anyone can openly access, JC advertised for workers aged 18 years and older who resided in the United States (thereby, creating a “closed” survey) and offered to pay them US $1 to complete the survey. By using MTurk, JC was unable to report how many potential people saw the advertised survey. The Institutional Review Board at Michigan State University determined that this research was exempt from full board review. Participants provided consent by answering a yes-no question at the start of the survey before they could move to the first question.

### Survey

The survey was administered in two parts. Prior to accessing the survey, participants read an informed consent statement that described that participation was voluntary and that they could stop at any time. By clicking on a “next” button, participants were informed that they were providing consent to complete the survey. The first part asked participants basic demographic characteristics including year of birth, which was used to determine age and generational membership (eg, baby boomers, Gen X [18]); education; sex; income; race; political party affiliation; and place of residence (US state). Age was included to determine differences in knowledge and behavioral patterns based on age. Some reports in the United States essentially callout different age groups for ignoring public health recommendations [7,19]. In addition, there are well described patterns of health literacy based on education level [20] and race [21], which may not be present in a homogeneous society such as China. Political party identification is associated with many attitudes and behaviors in the United States related to science and science-based recommendations [22,23]. Leaders from both major parties in the United States have reacted differently to the COVID-19 pandemic, likely influencing those who follow them [24,25]. No personal identifiers were collected.

The second part of the survey included 12 questions that were adapted from Zhong et al [14] to measure knowledge about COVID-19, including clinical characteristics, transmission, and prevention and control. The knowledge questions were scored with one point for each correct question, and an aggregate score was calculated (range 0-12), with higher scores indicating more knowledge about COVID-19. Three additional questions were asked to determine participation in specific behaviors related to recommendations from the CDC and the NIH, including whether participants had spent more money than usual in the last 2 weeks on cleaning supplies, personal hygiene products, and food (a proxy measure of hoarding); whether they had gone to any place in the last 5 days where there were more than 50 people present (contradicting CDC recommendations to avoid such gatherings); and if they had worn a mask when leaving the home in the last 5 days (contradicting CDC, NIH, and health care official guidance).

### Statistical Analyses

Sample characteristics were generated using frequency analysis and other descriptive statistics as appropriate (Table 1). Knowledge scores were compared using two-tailed independent sample *t* tests for differences in mean scores between males and females, as well as groups based on whether people had engaged in hoarding activity or not, had attended large gatherings or not, and had worn masks or not. In addition, two-tailed independent sample *t* tests were used to determine differences in mean age between people who had engaged in these activities or not. An analysis of variance was used to determine differences in mean knowledge scores among groups based on education, race, income, political party, and generational age groups (eg, baby boomers, Gen X; Table 2). A multivariable linear regression was used to determine which demographic characteristics influenced knowledge scores, and a binomial logistic regression was used to determine which characteristics influence participating in hoarding behavior, attending large group events, and using masks (Table 3). All analyses were conducted using SPSS (version 25; IBM Corp). Reporting results followed Checklist for Reporting Results of Internet E-Surveys (CHERRIES) guidelines [26].

The use of virtual private network networks allows people from all over the world to mimic US internet protocol (IP) addresses, so each participant was asked for their US state of residence, and this was compared to each IP address location to determine matches. JC then excluded responses from participants whose IP address location did not match their given state. A total of 36 participants were excluded for the final sample size of 1034. The survey was offered to MTurk workers on March 17, 2020, at 4:05 PM Eastern time, and all 1070 responses were completed by 6:13 PM Eastern time. To set the context for the setting of the study, at the time the survey was released, there were 5704 COVID-19 cases reported in the United States and 195,957 worldwide. At the date of this writing (March 24, 2020) there were 46,548 cases in the United States and 396,249 worldwide [27]. It is likely that these numbers vastly underrepresent the actual prevalence.

**Table 1:** Demographics and COVID-19 knowledge and behaviors of participants (N=1034).

Demographics		Participants	
Age (years), mean (SD)		37.11 (11.22)	
**Age categories, n (%)**			
	Baby boomers (born 1946-1964)		104 (10.06)
	Gen X (born 1965-1976)		140 (13.54)
	Millennials (born 1977-1995)		717 (69.34)
	Gen Z (born 1996 or later)		73 (7.06)
**Education, n (%)**			
	High school/general equivalency diploma		102 (9.86)
	Some college		295 (28.53)
	Bachelor degree		469 (45.36)
	Graduate/professional degree		168 (16.25)
**Race, n (%)**			
	White		784 (75.82)
	Black/African American		145 (14.02)
	Asian/Pacific Islander		69 (6.67)
	Other		36 (3.48)
Male sex, n (%)		602 (58.22)	
**Income (US $), n (%)**			
	0-29,999		232 (22.44)
	30,000-59,999		366 (35.40)
	60,000-89,999		235 (22.72)
	≥90,000		201 (19.44)
**Political party, n (%)**			
	Republican		289 (27.95)
	Democrat		487 (47.10)
	Independent		258 (24.95)
**Behaviors, n (%)**			
	Participant reported spending more money at a grocery or club store on cleaning supplies, personal hygiene products, or food than normal in the last 2 weeks		649 (62.77)
	Participant reported going to any place with more than 50 people in attendance at the same time in the last 5 days		320 (30.95)
	Participant reported wearing a mask when leaving home in the last 5 days		244 (23.60)
**Knowledge questions answered correctly, n (%)**			
	The main clinical symptoms of COVID-19^a^ are fever, fatigue, and dry cough (true).		948 (91.68)
	Unlike the common cold, stuffy nose, runny nose, and sneezing are less common in persons infected with COVID-19 (true).		668 (64.60)
	There currently is no effective cure for COVID-19, but early symptomatic and supportive treatment can help most patients recover from the infection (true).		942 (91.10)
	Not all persons with COVID-19 will develop severe cases. Those who are elderly and have chronic illnesses are more likely to be severe cases (true).		886 (85.69)
	Eating or contacting wild animals would result in infection by the COVID-19 virus (false).		541 (52.32)
	Persons with COVID-19 cannot transmit the virus to others when a fever is not present (false).		820 (79.30)
	The COVID-19 virus spreads via respiratory droplets of infected individuals (true).		917 (88.68)
	Ordinary residents can wear general medical masks to prevent infection by the COVID-19 virus (false; although this knowledge has changed since survey administration).		567 (54.84)
	It is not necessary for children/young adults to take measures to prevent infection with COVID-19 (false).		878 (84.91)
	To prevent infection with COVID-19, individuals should avoid going to crowded places and avoid public transportation (true).		973 (94.10)
	Isolation and treatment of people who are infected with COVID-19 are effective ways to reduce the spread of the virus (true)		957 (92.55)
	People who have contact with someone infected with the COVID-19 virus should be immediately isolated. In general, the observation period is 14 days (true).		955 (92.36)

^a^COVID-19: coronavirus disease.

**Table 2:** Group comparisons of knowledge scores and age comparisons of participants (N=1034).

Groups			Score, mean (SD)	*t* test/*F* test	*P* value
**Age categories**				*F*=9.184	<.001
	Baby boomers (born 1946-1964)		10.55 (1.48)		
	Gen X (born 1965-1976)		9.86 (1.74)		
	Millennials (born 1977-1995)		9.62 (1.94)		
	Gen Z (born 1996 or later)		9.19 (2.40)		
**Sex**				*t*=4.184	<.001
	Male		9.52 (2.07)		
	Female		10.01 (1.69)		
**Education**				*F*=7.513	<.001
	High school/general equivalency diploma		9.66 (1.96)		
	Some college		10.14 (1.49)		
	Bachelor’s degree		9.61 (2.01)		
	Graduate/professional degree		9.33 (2.24)		
**Race**				*F*=23.43	<.001
	White		9.92 (1.85)		
	Black/African American		8.51 (2.11)		
	Asian/Pacific Islander		9.91 (1.82)		
	Other		9.66 (1.39)		
**Income (US $)**				*F*=2.861	.04
	0-29,999		9.58 (1.85)		
	30,000-59,999		9.60 (2.13)		
	60,000-89,999		9.76 (1.83)		
	≥90,000		10.05 (1.73)		
**Political party identification**				*F*=21.821	<.001
	Republican		9.11 (2.07)		
	Democrat		10.04 (1.74)		
	Independent		9.79 (1.97)		
**Behaviors**					
	**Spent more money on cleaning supplies, personal hygiene products, or food than normal**			*t*=4.001	<.001
		Yes	9.54 (1.95)		
		No	10.02 (1.87)		
	**Participant reported going to any place with more than 50 people in attendance**			*t*=4.787	<.001
		Yes	9.26 (2.16)		
		No	9.93 (1.79)		
	**Participant reported wearing a mask when leaving home in the last 5 days**			===*t* =16.848	<.001
		Yes	8.02 (1.85)		
		No	10.25 (1.63)		
**Behaviors age comparisons**					
	**Spent more money on cleaning supplies, personal hygiene products, food than normal**			*t*=1.231	.22
		Yes	36.77 (10.42)		
		No	37.70 (12.47)		
	**Participant reported going to any place with more than 50 people in attendance**			*t*=1.895	.05
		Yes	36.18 (10.02)		
		No	37.54 (11.71)		
	**Participant reported wearing a mask when leaving home in the last 5 days**			*t*=4.153	<.001
		Yes	34.76 (9.63)		
		No	37.84 (11.58)		

**Table 3:** Determinants of knowledge score and behavior outcomes of participants (N=1034).

Groups		Knowledge score		Bought more goods, OR^a^ (95% CI)	Gathering of more than 50 people, OR (95% CI)	Wore mask, OR (95% CI)
		b (SE)	*P* value			
Constant, b (SE)		9.90 (0.29)	<.001	0.69 (0.51)	–0.08 (0.53)	2.99 (0.73)
R^2^		0.149	N/A^b^	0.08	0.07	0.45
Knowledge score		N/A	N/A	0.88 (0.81-0.95)	0.87 (0.81-0.93)	0.56 (0.50-0.62)
**Age (reference: baby boomers)**						
	Gen X (born 1965-1976)	–0.53 (0.24)	.02	1.76 (1.03-3.01)	1.23 (0.68-2.23)	1.28 (0.56-2.88)
	Millennials (born 1977-1995)	–0.64 (0.19)	.001	1.56 (1.01-2.41)	1.35 (0.82-2.22)	1.27 (0.63-2.54)
	Gen Z (born 1996 or later)	–1.28 (0.28)	<.001	0.94 (0.50-1.77)	0.96 (0.46-1.99)	0.83 (0.28-2.42)
Male sex		–0.31 (0.12)	.007	0.88 (0.67-1.15)	0.96 (0.73-1.28)	1.35 (0.92-1.96)
**Education (reference: high school/general equivalency diploma)**						
	Some college	0.36 (0.21)	.09	1.40 (0.88-2.23)	1.62 (0.93-2.81)	1.23 (0.51-2.95)
	Bachelor degree	–0.17 (0.20)	.39	1.88 (1.19-2.97)	1.59 (0.93-2.72)	4.47 (2.00-9.97)
	Graduate/professional degree	–0.41 (0.24)	.09	2.11 (1.22-3.65)	1.67 (1.46-4.87)	7.41 (3.07-17.9)
**Race (reference: white)**						
	Black/African American	–1.19 (0.17)	<.001	1.28 (0.84-1.95)	1.16 (0.78-1.73)	2.48 (1.52-4.07)
	Asian/Pacific Islander	–0.01 (0.23)	.97	1.45 (0.82-2.54)	0.93 (0.53-1.64)	0.62 (0.27-1.42)
	Other	–0.19 (0.31)	.54	0.80 (0.40-1.59)	1.11 (0.53-2.33)	1.40 (0.53-3.67)
**Income (US $; reference: 0-29,999)**						
	30,000-59,999	0.26 (0.15)	.09	1.44 (1.02-2.05)	1.13 (0.78-1.66)	0.99 (0.59-1.65)
	60,000-89,999	0.40 (0.17)	.02	1.44 (0.97-2.14)	1.04 (0.68-1.59)	1.21 (0.69-2.11)
	≥90,000	0.71 (0.18)	<.001	1.54 (1.01-2.36)	1.22 (0.78-1.90)	0.76 (0.42-1.39)
**Political party identification (reference: Republican)**						
	Democrat	0.76 (0.14)	<.001	1.07 (0.77-1.49)	0.70 (0.50-0.97)	0.52 (0.34-0.78)
	Independent	0.57 (0.16)	<.001	0.78 (0.54-1.12)	0.84 (0.58-1.23)	0.34 (0.19-0.57)

^a^OR: odds ratio.

^b^Not applicable.

## Results

A total of 1070 participants completed the survey. On average it took 4 minutes to complete the survey (equivalent to US $15/hour). Participants were on average 37.11 years of age ranging from 19 to 77. Of the 1034 participants, less than half of the participants completed a bachelor’s degree, more than three-fourths reported a white race, over half were male, over one-third reported an income between US $30,000 and US $59,999, and less than half identified as Democrats. Additional demographic information is included in Table 1.

Results for each of the COVID-19 knowledge questions are included in Table 1. Answers for questions ranged from over half to almost all participants answering correctly. The mean knowledge score was 9.72 (SD 1.93, range 0-12) for an overall correct percentage of approximately 80%, which was lower than the 90% correct rate that Zhong at al [14] reported in their sample of Chinese citizens at approximately 2 months into the outbreak.

Knowledge scores were significantly different between groups based on sex, generational ages, education, race, income, and political party identification. In general, baby boomers, females, those with some college education, and those with higher incomes were more knowledgeable about COVID-19, while black participants and Republicans were less knowledgeable (Table 2).

Regarding behaviors, participants who reported spending more money in the last 2 weeks, going to gatherings with more than 50 people, or wearing masks outside the home, were less knowledgeable about COVID-19 compared to participants who did not report these activities. In addition, participants who reported these behaviors were also significantly younger, except for increased spending, which had no significant difference in age (Table 2).

The multivariable linear regression (Table 3) results suggest several important relationships. First, compared to baby boomers, members of Gen X, millennials, and Gen Z had significantly lower COVID-19 knowledge scores. Exponentiating the unstandardized parameter estimate indicates that predicted mean knowledge scores for Gen X, millennials, and Gen Z were 42%, 53%, and 73%, respectively, lower than baby boomers. Second, black participants had mean knowledge scores that were 70% lower when compared to whites. Third, participants with higher incomes had higher knowledge scores. Fourth, Democrats and independents had mean knowledge scores that were 113% and 76% higher, respectively, than Republicans.

The binary logistic regression analysis (Table 3) results revealed several predictors of each behavior. Self-reports of buying more goods than usual was negatively associated with COVID-19 knowledge. For every point increase in knowledge score, the odds of reporting unusual buying behavior decreased by 12%. In the context of generational groups, the odds of reporting purchasing behavior increased by 76% and 56% for Gen X and millennials, respectively, compared to baby boomers. In addition, people with higher education were associated with increased buying behaviors. The odds of unusual purchasing behavior increased by 88% and 111% for people with bachelor’s degrees and graduate or professional degrees, respectively, compared to those with a high school education. Finally, those with higher incomes had increased odds of unusual purchasing behavior.

For every point increase in knowledge scores, the odds of attending large gatherings in the last 5 days decreased by 13%. Participants with graduate or professional degrees had 67% greater odds of attending large gatherings, compared to those with a high school education. Finally, Democrats had 30% lower odds of attending large gatherings compared to Republicans.

For every point increase in knowledge scores, the odds of wearing a mask outside the home decreased by 44%. The largest effect of any of the analyses revealed that those with a bachelor’s degree or a graduate or professional degrees had 347% and 641%, respectively, increased odds of wearing masks outside the home compared to respondents with a high school education. Black participants had 148% increased odds of wearing masks outside the home compared to white participants. In addition, Democrats and independents had 48% and 66% lower odds, respectively, of reporting wearing masks compared to Republicans.

## Discussion

The PUS literature posits that an increase in knowledge leads people to understand science and trust in the institution of science. Extending this to the current COVID-19 pandemic, JC hypothesized that increased knowledge should lead to willingness to follow public health recommendations. In this sample, lower knowledge is associated with self-reports of engaging in purchasing more goods than necessary, attending gatherings of more than 50 people, and wearing medical masks outside the house. In addition, there were differences in knowledge about COVID-19 based on age group. In fact, contrary to recent US media, baby boomers in this sample were more knowledgeable about COVID-19 than all other age groups and were less likely to engage in purchasing behavior that could be considered hoarding. In general, people who did not engage in these behaviors had significantly higher knowledge scores. Finally, people who reported attending large gatherings and wearing masks in public were younger on average.

The average knowledge score for this entire sample was about 9.72 out of 12 total points (approximately 80%); this was 8 weeks after the first case was diagnosed in the United States. Approximately 8 weeks after the first diagnosis in China, the mean knowledge score for a sample of Chinese citizens was 10.8/12 (approximately 90%) [14], and it was suggested that the knowledge of Chinese citizens was high because of their experiences with the severe acute respiratory syndrome outbreak in the early 2000s and the observation that this sample was relatively affluent and highly educated. In this study, a difference of 1 point (8%) on the knowledge test is equivalent to about one question. This is a small difference, and most of the differences detected as statistically significant were about 1 point or less between groups. The large sample size likely contributes to this observation, but much smaller sample sizes on the order of 50-100 could have also detected these differences as significant. In addition, the knowledge differences detected based on age, race, sex, and political ideology were in agreement with other literature about controversial scientific topics.

In this sample, nearly 30% of people reported attending gatherings or going to places with more than 50 people in the last 5 days, contrary to advice from the CDC since March 12, 2020 (survey conducted on March 17, 2020). In China, only 3.6% of people reported going to crowded places in the previous 2 weeks [14]. It is possible that the coordinated effort and unchecked authority of the Chinese government to lockdown provinces provided most of the motivation for Chinese citizens to obey these mandates. To date, there has not been a coordinated effort by the US government to lockdown the nation. There is some debate whether the federal government even has constitutional authority, so individual states are left to make decisions about “shelter at home” policies and similar efforts. As of this writing, California, Illinois, New York, Washington, Michigan, Massachusetts, Indiana, Oregon, and West Virginia have issued stay-at-home orders; however, no state had issued a stay-at-home order as of the date of the survey, March 17, 2020. California was the first state in the nation to issue the order on March 19, 2020. Although many citizens all over the country could have anticipated some of these stay-at-home policies, which might have led them to change their purchasing behaviors, it is not possible with this data to determine if there were differences in purchasing behavior based on the presence of a stay-at-home order. With about 1 in 3 US citizens ordered to stay home, it is likely in the coming weeks that fewer people will report attending large gatherings. With recent changes in recommendations about wearing masks, that number is also likely to change.

Use of masks is an evolving and cultural phenomenon. In Asia, people are encouraged and even mandated to wear masks outside the house. In China, only 2.0% of people reported not wearing masks outside the home [14]. In this sample, approximately 76% of people did not wear masks outside the home in the last 5 days, which is perhaps reflective of the CDC and NIH recommendations that the general public not use masks so that they are saved for frontline health care workers [28]. However, it is probably more likely that masks could not be found in the United States because of a lack of supply combined with hoarding behavior [10]. Still, 24% of people reported using masks, indicating that a large section of the US public chose to ignore recommendations. It is important to note that the debate on masks has changed even since this survey was conducted, and the nationwide recommendation now is to wear masks, which has been made based on the understanding that many people with mild symptoms may not even know they are infected with COVID-19. Mask use could prevent infecting others by asymptomatic carriers. Knowledge about COVID-19 is rapidly changing, and what was considered “correct” at the time of this writing may not be “correct” anymore.

Political party identification significantly influenced knowledge about COVID-19 as well as behaviors related to attending large gatherings and wearing medical masks. To summarize, Republicans had lower knowledge and had higher odds of attending large gatherings and wearing masks in public compared to Democrats and independents. These behaviors directly contradict recommendations by both the CDC and NIH. In the United States, there is a widening gap in trust in science and science-based recommendations based on political party [22], which may contribute to the observation here that Republicans are more likely to ignore recommendations about the COVID-19 response. In addition, the results reported here suggest that there continues to be political divisions over the role of scientific experts in policy matters [23]. That is, Democrats want expert involvement and believe scientists should be involved in policy recommendations. Conversely, Republicans believe scientists should stay out of policy debates. These attitudes may be reflected in the results that Republicans have lower knowledge about COVID-19 and have higher odds of participating in behaviors that are not recommended by authorities to stem the tide of the current pandemic. However, to more definitively conclude anything about the involvement of scientists in policy debates, specific questions about this matter could be added to future surveys.

There are some limitations to this research. First, knowledge questions were not validated and scientific knowledge is currently a moving target. For example, although the current consensus is that eating wild animals will not transmit the disease, living and working in close proximity to animals clearly influenced this outbreak and could influence future outbreaks. As such, the argument for banning wet markets in China is gaining momentum, but knowledge about proximity to animals, as opposed to using them as a food source, might be conflated. Second, knowledge regarding who is most at risk for COVID-19 may change as the pandemic proceeds, as well as with experiences in different countries. For instance, fewer younger people in China were infected, while in the United States, a different pattern appears to be emerging [29]. Third, this was a convenience sample of US residents from every state in the country, but people were able to self-select based on their interest and experience with the topic. It is possible that sample demographics may not completely represent the US public. Fourth, although the survey questions were not able to be validated given the fast-moving nature of the pandemic response in the United States, the questions do have face value in the context of the situation at the time the survey was conducted. However, the first question about purchasing behavior (cleaning supplies, hygiene products, and food) might be better asked as three separate questions. Fifth, the theory that PUS and knowledge about science drives behavior is just one theory to study behaviors, attitudes, and beliefs. Future studies could incorporate models based on the theory of planned behavior, social cognitive theory, or even health belief models with questions devised to elicit responses about how desires, needs, and beliefs drive the types of behaviors studied here.

This survey is one of the first attempts to describe determinants of US public knowledge and behavioral response to the emerging COVID-19 pandemic in the United States. Although knowledge about COVID-19 is generally high, there are differences in knowledge based on age, sex, education, income, race, and political party identification. These differences appear to have prevented a coordinated effort at slowing the spread of the pandemic in the United States in the early days of the pandemic. Ignoring official recommendations for crowd avoidance, the use of medical supplies, and purchasing behaviors that signal hoarding of goods, does not bode well for efforts to contain the spread of the virus and limit exposure to vulnerable populations. Without a coordinated national response, it is likely that the United States will experience a longer, more drawn out battle than if such coordination would occur. In addition, it is important for future waves of COVID-19 that we consider implementing specific policies and programs to target groups of people who have been unequally affected by the pandemic. Now is the time for policy makers to address the structural issues in many urban areas that adversely affect minority health care, especially when we observe disparities in mortality based on race. Now is the time for policy makers to ensure that access to care, especially specialized care, in rural areas does not hamper the response to COVID-19, which is likely to hit rural areas in the next wave. Finally, it is time for policy makers to reverse the decades long decimation of public health funding and infrastructure that has left the United States so vulnerable to the ravages of this pandemic.

## Abbreviations

CDC: Centers for Disease Control and Prevention
CHERRIES: Checklist for Reporting Results of Internet E-Surveys
COVID-19: coronavirus disease
IP: internet protocol
MTurk: Mechanical Turk
NIH: National Institutes of Health
OR: odds ratio
PUS: public understanding of science
SARS-CoV-2: severe acute respiratory syndrome coronavirus 2
